# Developing a predictive nomogram and web-based survival calculator for locally advanced hypopharyngeal cancer: A propensity score-adjusted, population-based study

**DOI:** 10.17305/bb.2023.8978

**Published:** 2023-10-01

**Authors:** Sihao Chen, Shanshan He, Dan Wang, Yi Liu, Shilong Shao, Li Tang, Chao Li, Qiuling Shi, Jifeng Liu, Feng Wang, Shichuan Zhang

**Affiliations:** 1 Department of Oncology, Affiliated Hospital of Southwest Medical University, Luzhou, China; 2 Department of Radiation Oncology, Sichuan Cancer Hospital and Institute, School of Medicine, University of Electronic Science and Technology of China, Sichuan Cancer Center, Radiation Oncology Key Laboratory of Sichuan Province, Chengdu, China; 3 School of Medicine, University of Electronic Science and Technology of China, Chengdu, China; 4 College of Public Health, Chongqing Medical University, Chongqing, China; 5 State Key Laboratory of Ultrasound in Medicine and Engineering, School of Public Health and Management, Chongqing Medical University, Chongqing, China; 6 Department of Otolaryngology, Head and Neck Surgery, West China Hospital, Sichuan University, Chengdu, China; 7 Department of Medical Oncology, Cancer Center, West China Hospital, West China Medical School, Sichuan University, Sichuan, China

**Keywords:** Locally advanced hypopharyngeal squamous cell carcinoma (LA-HPSCC), nomogram, survival, surgery-based therapy, Surveillance, Epidemiology, and End Results (SEER)

## Abstract

Understanding the clinical features and accurately predicting the prognosis of patients with locally advanced hypopharyngeal squamous cell carcinoma (LA-HPSCC) is important for patient-centered decision making. This study aimed to create a multi-factor nomogram predictive model and a Web-based calculator to predict post-therapy survival for patients with LA-HPSCC. A retrospective cohort study analyzing Surveillance, Epidemiology, and End Results (SEER) database from 2004 to 2015 for patients diagnosed with LA-HPSCC was conducted and randomly divided into a training and a validation group (7:3 ratio). The external validation cohort included 276 patients from Sichuan Cancer Hospital, China. The least absolute shrinkage and selection operator (LASSO)-Cox regression analysis was used to identify independent factors associated with overall survival (OS) and cancer-specific survival (CSS), and nomogram models and Web-based survival calculators were constructed. Propensity score matching (PSM) was used to compare survival with different treatment options. A total of 2526 patients were included in the prognostic model. The median OS and CSS for the entire cohort were 20 (18.6–21.3) months and 24 (21.7–26.2) months, respectively. Nomogram models integrating the seven factors demonstrated high predictive accuracy for 3-year and 5-year survival. PSM found that patients who received surgery-based curative therapy had better OS and CSS than those who received radiotherapy-based treatment (median survival times: 33 months vs 18 months and 40 months vs 22 months, respectively). The nomogram model accurately predicted patient survival from LA-HPSCC. Surgery with adjuvant therapy yielded significantly better survival than definitive radiotherapy and should be prioritized over definitive radiotherapy.

## Introduction

The hypopharynx is a transitional structure placed between the pharynx and cervical esophagus. Squamous cell carcinoma of the hypopharynx is usually diagnosed at an advanced stage and therefore has a very poor prognosis [1–3]. The treatment modalities for locally advanced hypopharyngeal squamous cell carcinoma (LA-HPSCC) include surgery, radiotherapy, and chemotherapy. However, the optimal therapeutic combination remains controversial [4, 5]. The VALGSG [6] and EORTC 24891 trials [7] have shown that induction chemotherapy followed by radiotherapy (IC + RT) can be used as a strategy for organ preservation without loss of survival. The RTOG 91-11 trial [8] further demonstrated that concomitant chemoradiotherapy (CRT) is superior to IC + RT for the laryngectomy-free survival in laryngeal cancer patients, with an equal overall survival (OS). Based on these high-level clinical evidence, IC + RT and concomitant CRT have been widely used as the frontline treatments for LA-HPSCC. However, real-world data on both laryngeal and hypopharyngeal carcinomas have raised concerns that organ-preserving treatment may be detrimental to patients’ OS [9–11]. Careful analysis of patient data is essential to address the contradictions between trials and real-world observations.

**Figure 1.**
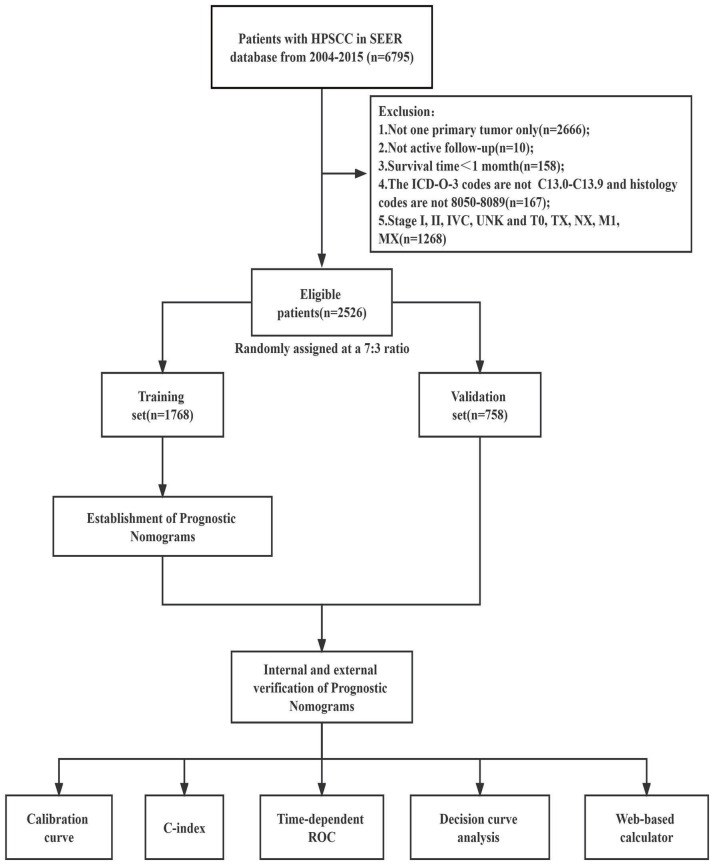
**Study design and the workflow diagram.** SEER: Surveillance, Epidemiology, and End Results; ROC: Receiver operating characteristic curve; C-index: Concordance index; HPSCC: Hypopharyngeal squamous cell carcinoma.

The Surveillance, Epidemiology, and End Results (SEER) database contains information on cancer diagnosis and different approaches used for the first course of cancer treatment. Several studies have analyzed the prognostic factors for LA-HPSCC using the SEER database, but inconsistent results have been generated [11–13]. In this study, we carefully selected patients who received definitive radiotherapy (radiotherapy-based) or radical surgery (surgery-based) from the SEER database from 2004 to 2015 and analyzed the factors with prognostic impact. Nomogram models integrating multiple prognostic factors were developed to predict the patient survival from LA-HPSCC [14]. The model was externally validated by an independent cohort of patients with LA-HPSCC from Sichuan Cancer Hospital, China.

## Materials and methods

### Data source and selection criteria

Screening criteria were as follows: (1) patients diagnosed with hypopharyngeal squamous cell cancer between 2004 and 2015, coded as C13.0, C13.1, C13.2, C13.8, or C13.9, according to the International Classification of Diseases for Oncology, Third Edition (ICD-O-3); (2) patients with histopathological confirmation of hypopharyngeal squamous cell cancer, coded as 8050-8089, according to the ICD-O-3; (3) patients with known survival time and > 1 month; (4) patients with complete follow-up data; and (5) patients with available detailed information on variables, including vital status, survival months, age, sex, race, T/N/M stage, and treatment mode of the primary tumor (Figure 1). Treatment data were extracted from the following fields: radiation sequence with surgery, reason for no cancer-directed surgery, radiation recovery, and chemotherapy recovery. Patients were included in the surgery-based therapy group if the surgery has been performed, including treatment categories of “radiation after surgery,” “radiation before and after surgery,” “radiation prior to surgery,” “sequence unknown, but both were given,” and “surgery both before and after radiation.” Patients were included in the radiotherapy-based therapy group if they received radiotherapy without surgery, including categories of “beam radiation,” “combination of beam with implantation,” and “radiation, NOS method, or source.” Patients without any records of radiation or surgery were included in the “chemotherapy/others” group (Figure S1). For external validation, we enrolled 276 patients with LA-HPSCC, who were treated at the Sichuan Cancer Hospital between January 2004 and June 2016.

### Ethical statement

The study was conducted in accordance with the Declaration of Helsinki (revised in 2013) and approved by the Institutional Review Board of the Sichuan Cancer Hospital (No. SCCHEC-02-2022-053). All data were de-identified, and the requirement for individual consent for this retrospective analysis was waived. This study followed the transparent reporting of a multivariable prediction model for individual prognosis or diagnosis (TRIPOD) reporting guidelines for prognostic studies. Patient data were retrieved from the updated SEER database (https://seer.cancer.gov). The SEER* Stat software (version 8.3.8; https://seer.cancer.gov/seerstat) was used to download the data.

### Statistical analysis

The overall population data extracted from the SEER database were randomly divided into the training and validation groups in a ratio of 7:3. All categorical variables were presented as frequencies and percentages and were analyzed using the Chi-squared test. Least absolute shrinkage and selection operator (LASSO)-Cox regression analysis was used to estimate the relationship between predictor variables and survival outcomes. Independent prognostic variables (*P* < 0.05) were then selected for the development of a prognostic nomogram. Survival analysis was performed using the Kaplan–Meier method and the log-rank test. Discrimination was quantified using the area under the time-dependent receiver operating characteristic curve (time-dependent ROC) and the concordance index (C-index). Calibration curves were constructed for the evaluation of the concordance between the predicted survival probability and the observed probability. The decision curve analysis (DCA) was performed to compare the predictive value between our model and the American Joint Committee on Cancer (AJCC) stage. Propensity score matching (PSM) was used to analyze the survival differences between the treatment modalities. All analyses were performed with the SPSS 26.0 and R software version 4.1.1 (https://www.r-project.org/), using the rms, timeROC, DCA, DynNom, and shiny packages. Statistical significance was set at *P* < 0.05.

## Results

### Characteristics of patients

The entire cohort from the SEER database included a total of 2526 LA-HPSCC patients that met the inclusion criteria from 2004 to 2015. The patients were randomly stratified into two groups in a ratio of 7:3, with 1768 patients in the training group and 758 patients in the validation group (Table 1). There was no statistical difference in the distribution of patients between the training and validation groups (*P* > 0.05). The demographic and clinical characteristics of patients are shown in Table 1. The majority of patients were men (*n* ═ 2084, 82.5%), white race (*n* ═ 1884, 74.6%), insured (*n* ═ 1787, 70.7%), and presented with lesions located in the pyriform sinus (*n* ═ 1377, 54.5%). Regarding treatment modalities, the percentage of patients who received surgery-based, radiotherapy-based, and chemotherapy alone or other treatments were 14.7%, 72.4%, and 12.9%, respectively. Additionally, 276 patients with LA-HPSCC who were treated at the Sichuan Cancer Hospital were enrolled for external validation. Their demographic and clinical characteristics are presented in Table S1. For the entire population in the SEER database, the median survival time was 20 (18.6–21.3) months. The median survival in the training group was 19 (17.4–20.5) months and in the validation group 21 (18.9–23.1) months. For the external validation group, the median survival time was 19 (15.1–23.1) months.

**Table 1:** Characteristics of patients with LA-HPSCC in the training and validation group

**Characteristics**	**Total (*n* ═ 2526)** ***n* (%)**	**Training group (*n* ═ 1768)** ***n* (%)**	**Validation group (*n* ═ 758)** ***n* (%)**	***P* value**
*Age (years)*				0.808
<65	1520 (60.2)	1057 (59.8)	463 (61.1)	
65–75	677 (26.8)	457 (25.8)	220 (29.0)	
76–85	279 (11.0)	218 (12.3)	61 (8.1)	
>85	50 (2.0)	36 (2.1)	14 (1.8)	
*Sex*				0.826
Male	2084 (82.5)	1468 (83.1)	616 (81.3)	
Female	422 (17.5)	300 (16.9)	142 (18.7)	
*Race*				0.975
White	1884 (74.6)	1311 (74.1)	573 (75.6)	
Black	459 (18.2)	326 (18.4)	133 (17.5)	
Others	183 (7.2)	131 (7.5)	52 (6.9)	
*Insurance*				0.987
Yes	1787 (70.7)	1245 (70.4)	542 (71.5)	
No/Unknown	739 (29.3)	523 (29.6)	216 (28.5)	
*Marital status*				0.684
Married	1083 (42.9)	737 (41.7)	346 (45.6)	
Others	1443 (57.1)	1031 (58.3)	412 (54.4)	
*Primary site*				0.997
Pyriform sinus	1377 (54.5)	965 (54.6)	415 (54.7)	
Postcricoid region	60 (2.3)	34 (1.9)	26 (3.4)	
Aryepiglottic fold	131 (5.3)	92 (5.2)	36 (4.7)	
Posterior wall	145 (5.7)	102 (5.8)	43 (5.7)	
Overlapping lesion	100 (3.9)	68 (3.8)	32 (4.2)	
NOS	713 (28.3)	507 (28.7)	206 (27.3)	
*Pathological grade*				0.589
I–II	1121 (44.4)	785 (40.8)	322 (42.5)	
III–IV	873 (34.5)	588 (33.5)	283 (37.4)	
Unknown	532 (21.1)	395 (25.7)	153 (20.1)	
*T stage*				0.531
T1	193 (7.6)	127 (7.2)	66 (8.7)	
T2	733 (29.1)	505 (28.6)	228 (30.1)	
T3	638 (25.2)	450 (25.5)	188 (24.8)	
T4a	693 (27.5)	492 (27.8)	201 (26.5)	
T4b	269 (10.6)	194 (10.9)	75 (9.9)	
*N stage*				0.976
N0	319 (12.6)	216 (12.2)	103 (13.7)	
N1	644 (24.5)	443 (25.1)	201 (26.5)	
N2	1401 (55.5)	992 (56.1)	409 (53.9)	
N3	162 (6.4)	117 (6.6)	45 (5.9)	
*AJCC stage*				0.972
III	588 (23.3)	405 (22.9)	183 (24.2)	
IVa	1548 (61.3)	1083 (61.3)	465 (61.3)	
IVb	390 (15.4)	280 (15.8)	110 (14.5)	
*Treatments*				0.906
Surgery-based	371 (14.7)	251 (14.2)	120 (15.8)	
Radiotherapy-based	1830 (72.4)	1286 (72.6)	544 (71.8)	
Chemotherapy or others	325 (12.9)	231 (13.2)	94 (12.4)	

LA-HPSCC: Locally advanced hypopharyngeal squamous cell carcinoma; NOS: Not otherwise specified; AJCC: American Joint Committee on Cancer.

### Screening of the independent prognostic factors

**Figure 2.**
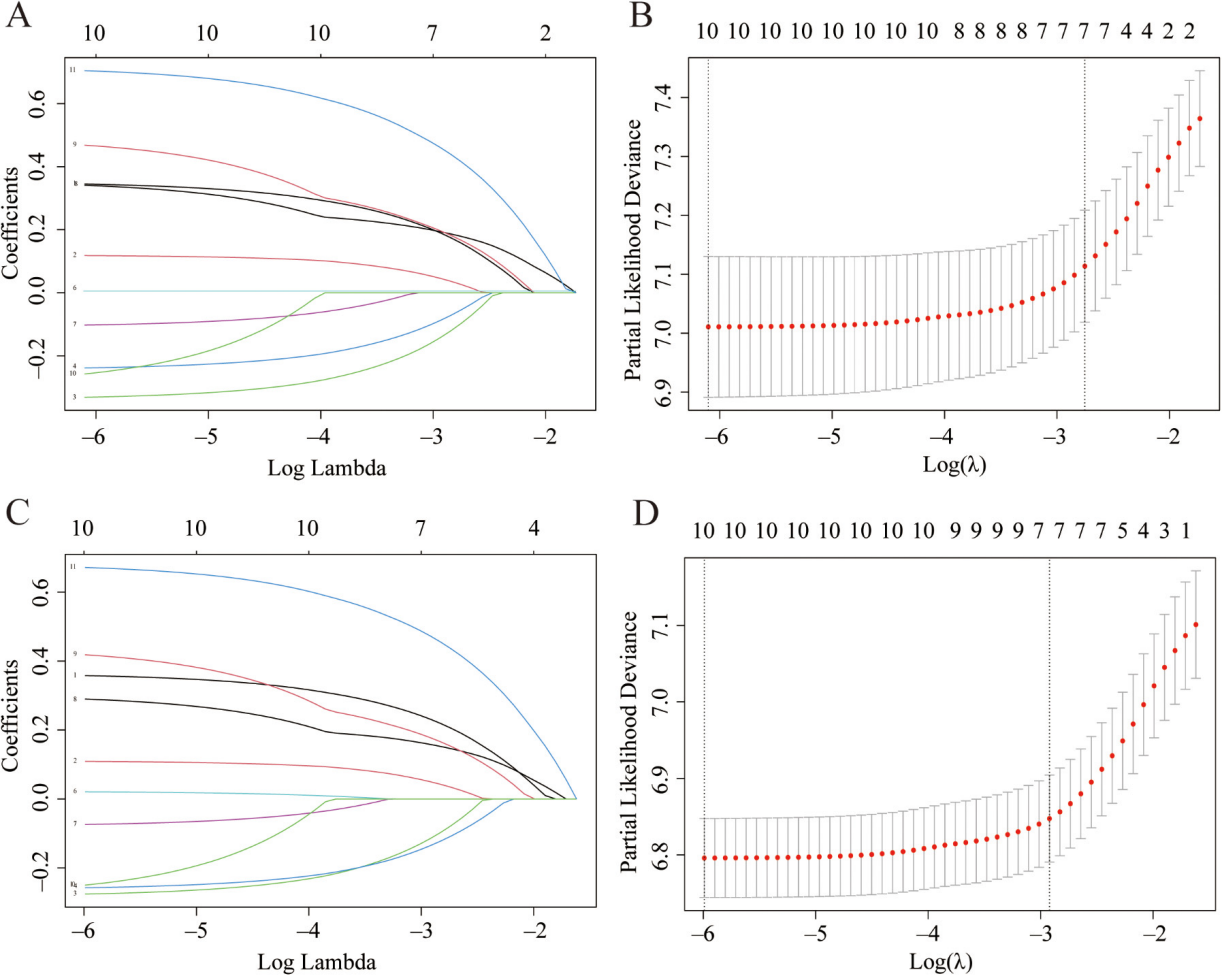
**Feature selection using the LASSO--Cox regression.** Profiles of LASSO coefficient for clinical and pathological features in OS (A) and CSS (C). Selection of tuning parameter (lambda) in the LASSO regression using 10-fold cross-validation in OS (B) and CSS (D). OS: Overall survival; CSS: Cancer-specific survival; LASSO: Least absolute shrinkage and selection operator.

To effectively avoid possible overfitting in the process of model screening variables [15], LASSO-Cox regression analysis was used to determine the optimal coefficient for each prognostic factor based on the smallest partial probability deviation and generate coefficient curves from logarithmic (lambda) series (Figure 2A and 2C). We found that age, race, insurance, marital status, T stage, N stage, and treatment were identified as the seven independent predictors in the OS and cancer-specific survival (CSS) models, according to the minimum requirements for LASSO-Cox regression analysis utilizing 10-way cross-validation (Figure 2B and 2D). The OS and CSS curves of the patients grouped by each predictor are shown in Figures S2 and S3.

### Construction and validation of the nomogram

**Figure 3.**
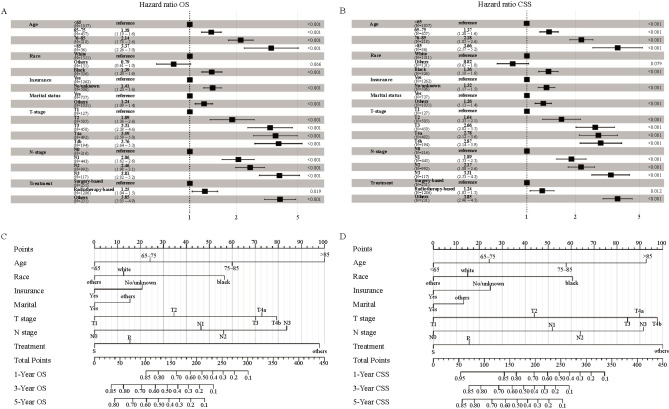
**Forest plot demonstrating the LASSO-Cox regression model for OS (A) and CSS (B) in the training cohort and the nomogram for predicting 1-, 3-, and 5-year OS (C) and CSS (D).** LASSO: Least absolute shrinkage and selection operator; OS: Overall survival; CSS: Cancer-specific survival.

The forest plot and prognostic nomograms integrating all significant independent factors in the training group are shown in Figure 3. The C-indices for OS and CSS prediction of the nomogram models for the training, internal validation, and external validation groups were greater than those of the AJCC stage, suggesting that the models had significantly higher predictive power for the AJCC stage of the disease (Table S2).

**Figure 4.**
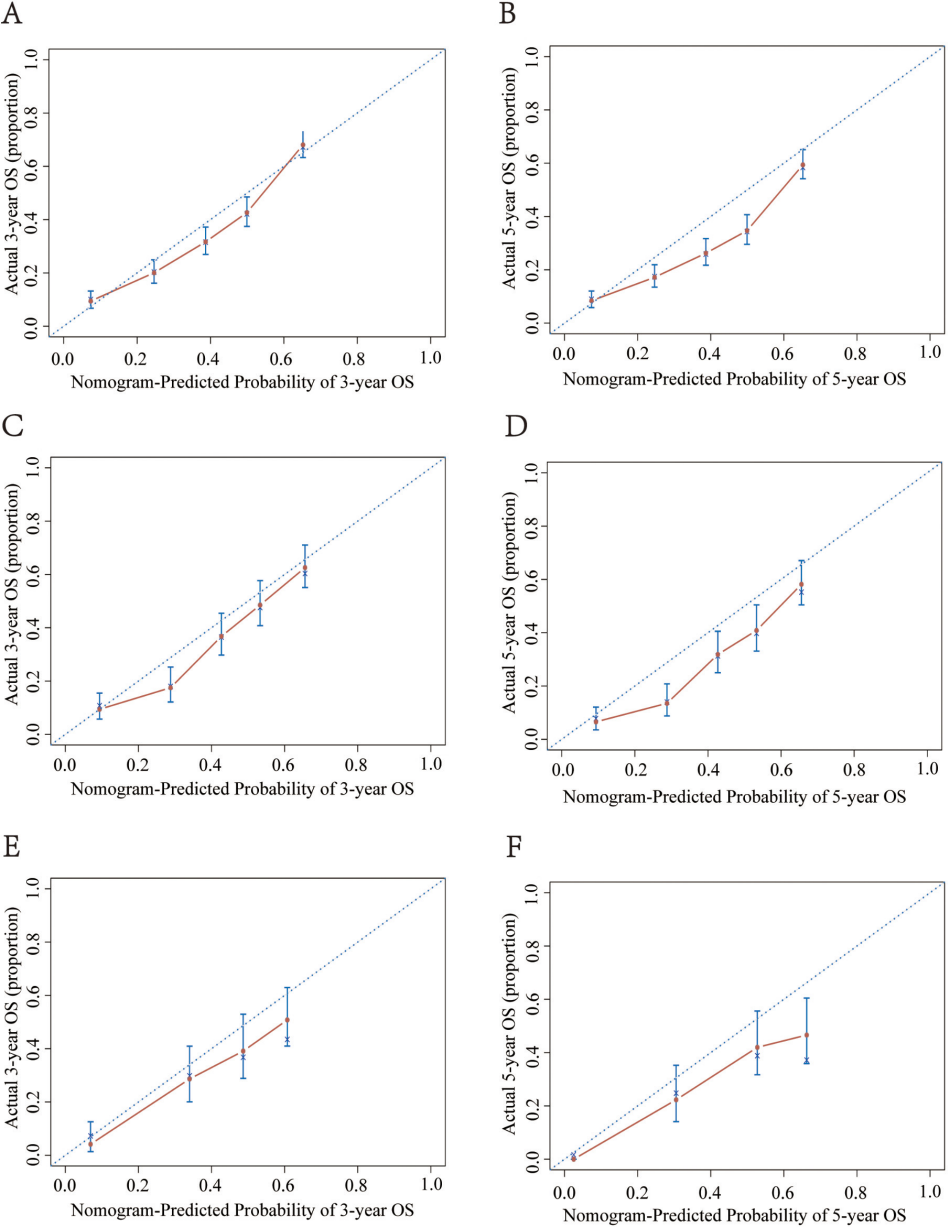
**The calibration curves predicting 3- and 5-year OS in the training (A and B), validation group (C and D), and external verification group (E and F).** OS: Overall survival.

**Figure 5.**
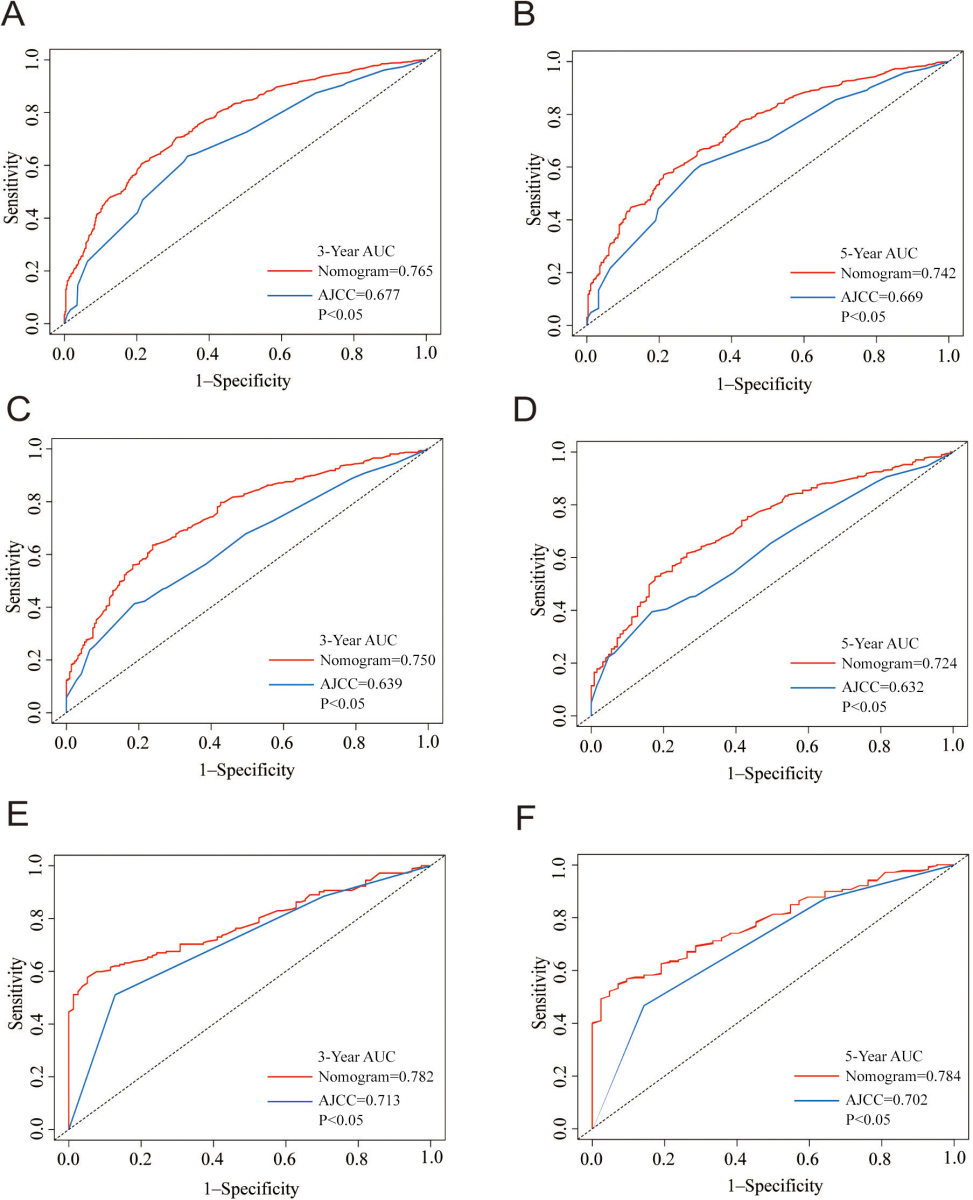
**The nomogram and the AJCC stage of the ROC curve analysis in the prediction of OS at the 3- and 5-year point in the training (A and B), internal validation (C and D), and external validation groups (E and F).** AJCC: American Joint Committee on Cancer; OS: Overall survival; AUC: Area under the curve; ROC: Receiver operating characteristic.

**Figure 6.**
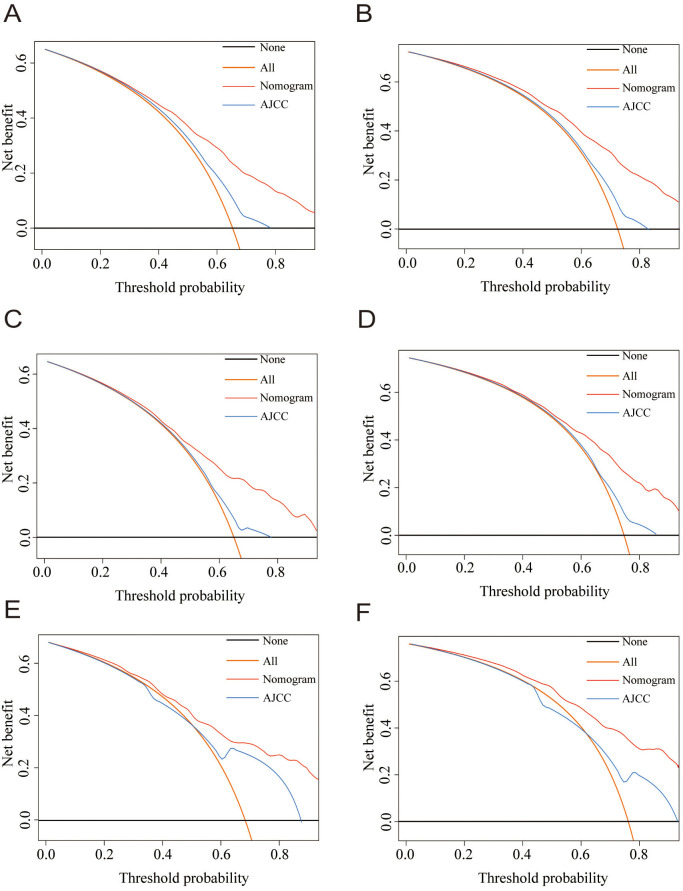
**The nomogram and the AJCC stage of the decision curve analysis in the prediction of OS at the 3- and 5-year point in the training (A and B), internal validation (C and D), and external validation groups (E and F).** AJCC: American Joint Committee on Cancer; OS: Overall survival.

The calibration plots showed consistency between predicted survival and actual survival. For the training, internal validation, and external validation groups, the nomogram models revealed good accuracy for the 3-year and 5-year OS (Figure 4) and CSS prediction (Figure S4). The ROC and DCA analyses are widely used validation methods for clinical predictive models, representing the overall accuracy and clinical applicability of the model, respectively [16, 17]. In this study, the ROC and DCA analyses both demonstrated that the models for OS (Figures 5 and 6) and CSS (Figure S5) were superior to the AJCC staging system in prognostic prediction.

### Development of an online survival estimate calculator

An online version of developed nomograms for OS and CSS in LA-HPSCC patients can be accessed at: https://la-hpscc.shinyapps.io/DynNomappHPSCC/ and /https://lahpscc.shinyapps.io/DynNomappHPSCCforCSS/ to further assist the researchers and clinicians. The predicted survival probability across time can be easily determined by inputting clinical features and reading output figures and tables generated by the Web server.

### Comparison between surgery-based and radiotherapy-based interventions and subgroup analysis

**Figure 7.**
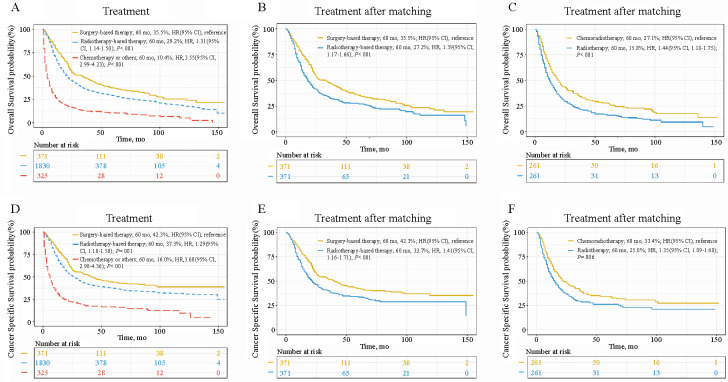
**Comparison of survival differences between treatment strategies analyzed by PSM.** Survival curves of groups before and after matching analysis in OS (A and D) and CSS (B, C, E, F). PSM: Propensity score matching; OS: Overall survival; CSS: Cancer-specific survival; HR: Hazard ratio; CI: Confidence interval.

To effectively control for confounding factors, the PSM analysis was used to compare the survival differences between the surgery-based and radiotherapy-based treatments [18]. Prior to the matched analysis, we observed that the surgery-based treatment had a better OS and CSS than the radiotherapy-based treatment, with median survival time of 34 vs 21 months and 42 vs 26 months, respectively (Figure 7A and 7D, and Table S3). After matching for patient characteristics, surgery-based treatment still provided a significant benefit in OS and CSS, with median survival time of 33 vs 18 months and 40 vs 22 months, respectively (Figure 7B and 7E, and Table S3). In addition, we also used PSM analysis to balance the clinical factors of patients treated with CRT and radiotherapy alone (Table S4) and found that adding chemotherapy significantly improved the patient survival rates (Figure 7C and 7F).

## Discussion

According to the SEER registration, there has been a significant shift from the surgery-based treatment to the radiotherapy-based treatment for HPSCC in the 1990s [19–21]. This shift is likely inspired by several organ/function preservation trials [22–25] on laryngeal and hypopharyngeal cancer that were published in the early 1990s. According to Hochfelder et al. and the current study, the latest status of treatment selection for LA-HPSCC was identified by analyzing the SEER records from 2004 to 2015 predominantly favored the radiotherapy-based treatment (4.3:1 in the Hochfelder study, 4.9:1 in the current study, and 4.4:1, if T4b excluded from the radiotherapy group). In an NCDB-based study [26], the ratio was as high as 7.6:1. Given the great change in treatment strategy, the survival of patients with LA-HPSCC remains poor, with a median survival of 20 months in the real world, reminding us that optimizing the current treatment setting and exploring new strategies to improve patient survival is the top priority of LA-HPSCC treatment, rather than pursuing organ preservation.

It has already been questioned whether organ preservation treatment sacrifices patient survival both in laryngeal cancer and HPSCC [27]. Hochfelder et al. performed the most comprehensive survival analysis of LA-HPSCC based on the SEER database. They concluded that surgery with adjuvant radiotherapy/CRT (S+Adj) provides significant benefits for both OS (hazard ratio [HR] 0.70, 95% confidence interval [CI] 0.59–0.84) and CSS (HR 0.66, 95% CI 0.54–0.82) compared with CRT. After adjustment, S+Adj was associated with a longer CSS than CRT (HR 0.75, 95% CI 0.57–0.99). However, this change was not observed with OS (HR 0.82, 95% CI 0.66–1.04). This study has retrieved the SEER database within the same period as the Hochfelder’s study but applied a new criterion for treatment classification. Patients who received monotherapy were excluded from the Hochfelder’s study but were included in this study. Local resection with radiotherapy was recognized as S+Adj in the Hochfelder’s study, but it was placed into the radiotherapy-based group in the current study because radiotherapy is considered as a definitive treatment in this setting. In the current study, only pharyngectomy and procedures beyond that (primary surgery codes 30 and 30+) were identified as radical surgery and thus classified as surgery-based treatment. Finally, we screened 371 patients who underwent radical surgery and confirmed that surgery-based treatment was superior to radiotherapy-based treatment in terms of both OS (HR 0.763, 95% CI 0.664–0.877, *P* < 0.001) and CSS (HR 0.775, 95% CI 0.663–0.906, *P* ═ 0.001). Notably, after adjusting for all baseline data, the differences remained (OS: HR 0.715, 95% CI 0.601–0.851, *P* < 0.001; CSS: HR 0.708, 95% CI 0.584–0.859, *P* < 0.001).

Sanabria et al. [27] have given an in-depth discussion on the survival gap between organ preservation trials and real-world outcomes. In brief, the trials enrolled patients according to strict criteria, whereas in the real world, radiotherapy may be recommended with loose judgment. The quality and timing of treatment were ensured in trials, whereas in the real world, both CRT and surgery may not have been of the same quality as observed in teaching hospitals, and salvage surgery might be delayed or not even offered. The authors concluded that in a non-academic setting, patients with T4 stage should receive total laryngectomy, whereas patients with T3 receive a function preservation strategy with all resources guaranteed. Their discussion mainly focused on laryngeal cancer, but it also included HPSCC. However, the prognosis of HPSCC is much worse than that of other head and neck squamous cell carcinomas, including laryngeal cancer [28, 29]. The recommendation of CRT to patients with advanced HPSCC thus should be more cautious, and definitive radiotherapy should be applied with closer observation than that for other tumors. Indeed, in the EORTC 24891 trial [7] designed specifically for LA-HPSCC, only 61.8% (60 of 97) of patients in the induction arm received definitive radiotherapy, with a radiotherapy/surgery ratio of approximately 1.8:1, a more stringent entry into radiotherapy than the real world.

Based on a detailed analysis of the records in the SEER database, the current study built a multidimensional predictive model for both OS and CSS. In addition to TNM staging, factors including age, race, insurance, marital status, and importantly, treatment approach, should all be considered when making treatment decisions. The models were validated not only by the internal cohort but also externally by the cohort of patients treated in China. To facilitate a comparison of the impact of different clinical factors, we created the OS and CSS online calculators that can be easily accessed and provide quick estimation of survival.

As mentioned above, even if radical surgery with adjuvant treatment is provided, the survival rate of LA-HPSCC remains poor. A new strategy that can efficiently combine chemotherapy, radiotherapy, and surgery requires further investigation. Case in point, the alternating chemotherapy and radiotherapy regimen proposed in the EORTC 24954 study [30] showed an equal OS and a prolonged but nonsignificant functional larynx survival compared with concurrent CRT. Additionally, a study from the Cancer Hospital Chinese Academy of Medical Sciences [31] (CHCAMS) adopted a response-adapted strategy based on an early response to concomitant CRT. This strategy yielded a significantly better 5-year OS and 5-year survival with a functional larynx than the primary RT group, with no additional operative complications. These two non-conventional treatment regimens may need more consideration than the conventional IC or CRT regimens. Immunotherapy using immune checkpoint inhibitors (ICIs) is a novel treatment that has greatly changed the field of oncology. Concomitant regimens that included ICI [32] for the treatment of head and neck cancer failed to challenge the standard care, whereas ICI in the induction setting [33–35] provided a promising prospect in treating local-regional advanced head and neck cancer.

One of the major limitations of the current study is that the SEER registration missed a few important bioinformatics and clinical factors, including performance score, details of chemotherapy (cycles and regimens), radiotherapy technique, dose, and target definition, time between diagnosis and the onset of therapy, targeted therapy/immunotherapy, and second-line treatment. The lack of these data may account for the lower accuracy of the 5-year survival prediction of the model. In addition, extra-nodal invasion has been found to be an important prognostic factor and is grouped into N3 stage in the 7th AJCC staging system [36]. This pathological indicator for poor prognosis was not recorded before 2015 in the SEER database, and the models may overestimate the survival of patients with extranodal invasion.

## Conclusion

Through a detailed analysis of patient records in the SEER database between 2004 and 2015, we found that multiple factors have an independent impact on the OS and CSS in patients with LA-HPSCC. Compared with the 72.4% (1830 in 2526) of patients who received radiotherapy-based treatment, only approximately 14.7% (371 in 2526) received surgery-based treatment. However, patients who received surgery-based treatment had significantly better survival (median survival time: 34 vs 21 months) and a reduced HR in OS (HR 0.763; 95% CI 0.664–0.877, *P* < 0.001). The current study strongly suggests that CRT should be recommended with caution for patients with LA-HPSCC. We provided online calculators for projecting the survival with different treatment modalities under given clinical conditions, which will be helpful for the physicians and patients to understand the prognosis and thus make treatment decisions accordingly.

## Supplemental Data

Supplementary data are available at the following link: https://www.bjbms.org/ojs/index.php/bjbms/article/view/8978/2763.
